# Developing a Return to Work Intervention for Breast Cancer Survivors with the Intervention Mapping Protocol: Challenges and Opportunities of the Needs Assessment

**DOI:** 10.3389/fpubh.2018.00035

**Published:** 2018-02-23

**Authors:** Jean-Baptiste Fassier, Marion Lamort-Bouché, Guillaume Broc, Laure Guittard, Julien Péron, Sabrina Rouat, Julien Carretier, Béatrice Fervers, Laurent Letrilliart, Philippe Sarnin

**Affiliations:** ^1^UMRESTTE UMR T_9405, Unité mixte de recherche Epidémiologique et de Surveillance Transport Travail Environnement, Université Claude Bernard Lyon 1, Université de Lyon, Lyon, France; ^2^Service de médecine et santé au travail, Hospices civils de Lyon, Lyon, France; ^3^Collège universitaire de médecine générale, Université Claude Bernard Lyon 1, Université de Lyon, Lyon, France; ^4^HESPER, Health Services and Performance Research, Université Claude Bernard Lyon 1, Université de Lyon, Lyon, France; ^5^Pôle IMER, Hospices civils de Lyon, Lyon, France; ^6^Laboratoire de Biométrie et Biologie Evolutive LBBE – UMR 5558, Université de Lyon, Université Claude Bernard Lyon 1, Lyon, France; ^7^Service d’oncologie médicale. Institut de Cancérologie des Hospices Civils de Lyon (IC-HCL), Hospices civils de Lyon, Pierre-Bénite, France; ^8^GRePS – EA 4163 (Groupe de Recherche en Psychologie Sociale), Université Lumière Lyon 2, Université de Lyon, Lyon, France; ^9^Centre Léon Bérard, Département Cancer et Environnement, Lyon, France; ^10^Faculté Lyon Est, Université Claude Bernard Lyon 1, Université de Lyon, Lyon, France

**Keywords:** intervention mapping, breast cancer, needs assessment, return to work, work rehabilitation, program development, participative research, social health disparities

## Abstract

Return to work (RTW) is an important step for breast cancer survivors (BCSs). However, they face many barriers that affect particularly women with low socioeconomic status (SES). Health care, workplace, and insurance actors lack knowledge and collaborate poorly. No intervention to date has proven effective to reduce social disparities in employment after breast cancer. The intervention mapping (IM) protocol is being used in France to develop, implement, and evaluate an intervention to facilitate and sustain RTW after breast cancer [*FAciliter et Soutenir le retour au TRAvail après un Cancer du Sein* (FASTRACS) project]. The research question of this study was to elicit the needs for RTW after breast cancer from various stakeholders’ point of view. The aim of this study was to describe the process and the preliminary results of the needs assessment of the FASTRACS project. *Different methods* were followed to (a) establish and work with a planning group and (b) conduct a needs assessment to create a logic model of the problem. A planning group was organized to gather the stakeholders with the research team. A review of the literature and indicators was conducted to identify the magnitude of the problem and the factors influencing RTW. A qualitative inquiry was conducted with 12 focus groups and 48 individual semi-structured interviews to explore the needs and experience of the stakeholders. The *results* of these tasks were the proposition of a charter of partnership to structure the participative process, a review of the scientific evidence and indicators, and the description by the stakeholders of their needs and experience. Many stakeholders disagreed with the concept of “early intervention.” They advocated for a better support of BCSs during their RTW, emphasized as a process. Anticipation, intersectoral collaboration, and workplace accommodation were mentioned to fit the needs of the BCS and their environment. A logic model of the problem was elaborated from these data. The ability of the model to consider specific characteristics of women with low SES is discussed, with a view to developing the FASTRACS intervention through the next steps of the IM protocol.

## Introduction

Breast cancer is the most common cancer in women worldwide (1), representing about 12% of all new cancer cases and 25% of all cancers in women. In France, an estimated 53,000 new cases were diagnosed in 2015 (2). Prognosis is good, with a standardized survival rate of 87% at 5 years from diagnosis and an estimated partial 5-year prevalence of 219,756 cases (2). Returning to work after a cancer is paramount, enhancing quality of life and financial independence and decreasing social costs (3). As the legal retirement age is to be postponed in many industrialized countries, the issue of return to work (RTW) after breast cancer is likely to affect a growing number of women worldwide.

The job status of French patients 2 years after the diagnosis of cancer is poor, with a decrease in the activity rate from 88.2 to 79.9% between 2010 and 2012 (4). In the Netherlands, the proportion of employed women who fully resumed working after breast cancer within 1 year of diagnosis has decreased, particularly in women over 50 years (from 59% in 2002 to 46% in 2008) (5). In the UK, a higher RTW rate (84%) was observed in the first year following treatment for breast cancer of health-care workers (6). A meta-analysis of 26 international studies from the US, Europe, and other countries showed that the unemployment rate is higher after breast cancer than after other cancers (35.6 vs 31.7%) (7). Even for women who succeeded in returning to work, a number of them still experience negative feelings while at work (8).

In the US, specific needs have been identified for certain categories of patients according to their race or income (9, 10), revealing social disparities in work resumption after breast cancer. In France, low-skilled women are more likely to lose their job 2 years later if they do not have job accommodation, whereas women with a management position are more likely to lose their job if they perceive a feeling of discrimination (4). In Denmark, a low socioeconomic status (SES) was identified as a risk factor for unemployment after breast cancer (11).

Although a fair amount of descriptive knowledge is available, intervention studies aimed at improving RTW after breast cancer have failed to prove their effectiveness (3). Despite the evidence of social disparities in employment after breast cancer, no intervention took into account their possible mechanisms which complexity needs clarification before proposing appropriate interventions (12, 13). A number of factors associated with a low social position are likely to act as causal and/or mediating factors of unemployment after breast cancer (heavy physical workload, low job latitude, temporary work contract, non-take-up of social rights, etc.). This might partially explain the failure of these interventions, which has also been attributed to the lack of conceptualization and the overmedicalization of a complex intersectoral issue (14). A recent review of 16 interventions addressing RTW after breast cancer showed that only one intervention referred to a theory linked to RTW (15). More than 80% of the interventions were provided by health-care professionals, and only 38% of the interventions were work-directed and offered other activities, such as coordination of services and information, as well as instructions for drawing up an RTW plan (15).

Furthermore, the implementation and routinization (sustainability) of interventions aimed at reducing social disparities usually fail to take into account the cultural and social specificities of the population they consider (16). In the field of complex interventions, it is therefore recommended to ensure a proper vision of the problem (theory of the problem) and of its solution (theory of action), not only from a scientific evidence-based point of view but also from the experience-based point of view of the actors in the field (target population and its environment) (17). The same has proven to be true in the specific field of work disability prevention, where it is recommended to base interventions upon explicit theories, in order to figure out their effective components with their relevant outcomes (14). It is also recommended to anticipate from the beginning the issues of implementation and sustainability, by involving the relevant categories of stakeholders (18, 19).

The intervention mapping (IM) protocol has been used for 15 years in different countries to develop, implement, and evaluate interventions in the field of health promotion (20, 21). It has been specifically used in cancer (22–24) and RTW (22, 25, 26). It develops a participative approach involving all the relevant stakeholders. It resorts to the theoretical frameworks in human and social sciences and requires a critical appraisal of the scientific literature (so-called evidence). Last but not least, the IM protocol relies on a global vision of the determinants of health, adopting an ecological perspective on the individuals within the different levels of their environment (interpersonal, organization, community, society, etc.) (16). In this respect, the IM protocol is acknowledged as an appropriate process to develop population-level health interventions likely to shift the distribution of health risk by addressing the underlying social, economic, and environmental conditions (27).

In view of its ability to help researchers face both theoretical and implementation issues in program development, the IM protocol was chosen in France by a research team in the frame of the *FAciliter et Soutenir le retour au TRAvail après un Cancer du Sein* (FASTRACS) project. The overall aims of the FASTRACS project are to develop, implement, and evaluate an intervention intended to facilitate and sustain RTW after breast cancer at a regional scale.

The aim of this study was to describe the process and the preliminary results of the needs assessment of the FASTRACS project during the first step of the IM protocol.

## Materials and Methods

### Study Setting

The FASTRACS project is led by a multidisciplinary research team associating a skill mix in psychology (health psychology, social psychology, and work psychology) and medicine (occupational medicine, general medicine, oncology, and public health) from the University of Lyon, in the Auvergne-Rhône-Alpes region of France. The scale of the project is to develop, implement, and test a pilot intervention at the county scale of “Metropole de Lyon” (59 cities with 1,280,000 inhabitants distributed over 538 km^2^). The strategic perspective at 5 years is to scale up and adapt the intervention in other counties of the same region if it is proven to be effective.

### Needs Assessment Tasks and Methods

According to the IM protocol (21), the learning objectives and tasks of the first step of the protocol are (a) to establish and work with a planning group; (b) to conduct a needs assessment to create a logic model of the problem; (c) to describe the context for the intervention, including the population, setting, and community; and (d) to state program goals. The present study will focus on the two first tasks.

#### Planning Group

Members of the research team liaised with key stakeholders, defined as field actors or members of institutions involved in RTW after cancer on a regular basis, knowledgeable, influential, and eager to commit themselves in a working group on a sustainable basis. First, a theoretical sampling was followed according to the arena model in work disability prevention (28), from which four categories of stakeholders were identified: patients and associations, health-care professionals and facilities, workplaces, and regional institutions representing the government, the social insurance system, and organizations involved in work disability prevention and handicap. Second, a purposeful and snowball sampling was followed from the personal network of the researchers to select the members of the planning group.

Two meetings were organized with the research team and the 25 members of the planning group. The first meeting was the occasion to present each other, to give an overview of the FASTRACS project and the IM protocol, and to establish the basis of a charter of partnership. During small group discussions, participants were asked to answer the following three questions: (a) What are the most important values to me (as an individual and/or for my institution)?”; (b) “What do I need (as an individual and/or for my institution) in order to collaborate in the FASTRACS project?”; and (c) “What commitment(s) can I take in the FASTRACS project (as an individual and/or for my institution)?” The participants produced written answers which content fed the discussion during the meeting. Their qualitative content analysis performed afterward structured the draft for the charter of partnership. A second meeting was organized 1 year later to share preliminary results of the data analysis of the needs assessment qualitative inquiry (see below).

#### Needs Assessment: Literature Reviews

A first literature review was performed up to June 2013 and regularly updated. Its aims were to gather the evidence from empirical studies about the occupational prognosis, the effectiveness of RTW interventions, and the lived experience of RTW of BCS. A sensitive search strategy was adopted to explore Medline, Healthstar, and Web of Science databases with the search string: [breast cancer AND (work OR employ$)].ti. In order to be included, the studies had to fulfill the following criteria: (a) deal with any aspect of RTW and job retention, (b) after breast cancer, and (c) provide information about the occupational prognosis (cohort studies), the lived experience reported by the patients or other stakeholders (qualitative studies) or interventions developed to help them RTW (intervention studies). Discussion papers and studies exploring (occupational) risk factors of breast cancer were excluded. Surveys were considered on an individual basis depending on their interest for the research topic. A priority was given to literature reviews before considering original studies. Another systematic literature review was performed to identify and describe the content of the interventions developed with the IM protocol in the field of cancer (29).

#### Needs Assessment: Review of Indicators

Indicators were searched in the scientific and the gray literature to document the extent and the scope of RTW issues after breast cancer in France (incidence of breast cancer, occupational consequences, comorbidities, etc.). Indicators in the scientific literature were identified from the literature review. The gray literature was identified from 10 different websites of the French national cancer institute (Institut National du Cancer), French cancer patient associations (Ligue contre le cancer and Europa Donna), and databases and websites of the Ministry of Health (Score-santé database, etc). From these websites, six reports were selected with relevant indicators.

#### Needs Assessment: Qualitative Inquiry

A qualitative inquiry was conducted among the four categories of stakeholders (patients, health care, workplaces, and institutions) aimed at exploring in-depth and in context their perceived needs and field experience. The data collection proceeded by 12 focus groups (FGs) and 48 individual semi-structured interviews, according to interview guides. The guides’ themes were based on the findings of the literature review and the clinical experience of the researchers. Some themes were common to each category of stakeholders to allow comparisons, and some other themes were specific to the stakeholder’s category to explore its specificities. The participants were identified through the personal network of the researchers and the members of the planning group who helped access both field actors and members from institutions. The sampling method was the same as the one used to select the members of the planning group. Respondent characteristics are described in Table 1. Themes and location of the interviews and FGs are presented in Table S1 in Supplementary Material.

**Table 1:** Respondent characteristics and data collection mode.

Stakeholder’s category	Respondent characteristics	Data collection mode	

		Focus groups (participants)	Interviews
**Breast cancer patients**			
	Women participating in a physical activity program after radiation therapy	3 (22)	10

**Workplace actors (7 workplaces; 5 private/2 public sector; and 1 small, 3 medium, and 3 large size companies)**			
	Former patients interviewed in their workplace		8
	Human resource directors		5
	Frontline managers		4
	Colleagues		5

**Health-care professionals**			
	General practitioners	3 (19)	
	Rehabilitation teams	3 (21)	
	Oncologists		20

**Institutions**			
	Social workers	2 (12)	5
	Insurance physicians	1 (4)	1

**Total**		12 (78)	48

### Data Analysis and Integration

The indicators retrieved from the scientific and the gray literature were categorized with an Excel™ spreadsheet. According to the IM protocol, indicators were sorted in the following categories: quality of life, health, and (occupational) environment.

For the qualitative inquiry, all interviews and FG meetings were recorded, transcribed, and anonymized (names of places and persons replaced by codes). The MAXQDA™ v11 and v12 software was used to conduct thematic qualitative content analysis. The analysis was performed by the researchers who conducted the interviews and FGs and the students they supervised. They were performed by different researchers investigating each stakeholder’s category, i.e., the breast cancer patients (Marion Lamort-Bouché, Jean-Baptiste Fassier, Philippe Sarnin, and Guillaume Broc), the workplace actors (Philippe Sarnin and Sabrina Rouat), the health-care practitioners (Marion Lamort-Bouché, Jean-Baptiste Fassier, and Guillaume Broc), and members from institutions (Jean-Baptiste Fassier).

In a first step, the themes of the interview and FG guides were used as coding categories with a deductive perspective. In a second step, new categories were created to analyze the data which did not fit in the initial coding tree and to refine the analysis of the data in an inductive perspective.

### Ethics

The needs assessment of the FASTRACS project received an ethical approval from the Comité de Protection des Personnes Sud-Est II (IRB no 00009118). All members of the planning group and participants to the qualitative needs inquiry received an information letter and signed a consent form.

## Results

### Planning Group

The first task resulted in establishing a planning group representing the wide diversity of stakeholders involved in RTW of employees with cancer (Table 2).

**Table 2:** Composition of the planning group.

Stakeholder’s category	Affiliation	Number of participants
**Patients and associations**		
	Europa Donna (breast cancer patients association)	2
	Ligue contre le cancer (cancer patients association)	1
	Juris Santé (association promoting the rights of patients)	1
	Individual patient	1

**Workplaces**		
	Food retailer, private employer, and 6,000 supermarkets in France	1
	Public university hospitals of Lyon, public employer, and 23,000 workers	1
	Pharmaceutical industry, private employer, and 250 workers	1
	Insurance company, private employer, and 24 workers	1
	Ventilation, heating, and cooling, private employer; and 1,400 workers	1

**Health-care professionals and organizations**		
	General practitioner (private practice)	1
	Occupational physician (private employers)	1
	Medical oncologist (public hospital)	1
	Radiation oncologist (private hospital)	1
	Rehabilitation medicine (public hospital)	1
	Nurse manager (public hospital)	1

**Institutions**		
	Regional cancer agency (Cancéropole CLARA)	1
	Metropole de Lyon (County health administration)	1
	Health insurance (regional agency, CARSAT) – insurance physician	1
	Health insurance (regional agency, CARSAT) – social worker	1
	Health insurance (regional agency, CARSAT) – prevention engineer	1
	Regional health agency (Agence régionale de santé)	2
	Regional work administration (DIRECTTE) – occupational medicine inspectorate	1
	Local agency for job retention of handicapped workers	1

Total		25

Regarding the charter of partnership, the qualitative content analysis of the written report of the first meeting of the planning group allowed identifying common themes (values, needs, and commitments) shared by the different stakeholders and by the research team members. The detailed process and results of the charter of partnership will be published. A comparative table of the values, needs, and commitments of the different stakeholders is provided in Table S2 in Supplementary Material. Shared values were expressed in terms of “respect” (mutual trust, non-judgmental attitude, and confidentiality) both within the research project and toward future participating patients, “solidarity” (brotherhood, equity, and attention to social disparities), “employment” (sustained employment, healthy jobs, and work as a determinant of health), “scientific rigor”, and “patient-centered program development”. Shared needs were expressed in terms of “communication” (need for a clear and open communication from the research team) and “partnership” (mutual acknowledgment, equity, and shared decisions). Shared commitments were expressed in terms of “personal commitment” (personal network and experience), “communication” (about the project), and “time” (participate on a sustainable basis).

### Needs Assessment: Literature Reviews

The first literature search in 2013 yielded 569 references from which 295 duplicates were removed. The remaining 274 records were screened and 213 were excluded on the basis of their title or abstract. Studies considered initially comprised cohort (*n* = 16) or register-based studies (*n* = 4), qualitative studies (15 original studies and 2 literature reviews), intervention studies (4 original studies and 3 literature reviews), and 17 surveys. This initial search was updated on a continuous basis with automatic alerts. The final evidence base comprised a number of original studies included in systematic reviews of cohort studies reporting on the occupational prognosis after breast cancer (30–32), reviews of qualitative studies reporting on the lived experience of breast cancer patients (33–35), and reviews of intervention studies aiming at RTW after breast cancer (3, 15, 36–38).

#### Factors Affecting RTW Rate and Time

According to cohort studies, factors affecting RTW rate and time are disease-related (prognosis, treatment, and symptoms), work-related (physical and psychological demands, social support at work), and social and demographic (age, education, and income level) (9, 39–42). Disease-related factors are fatigue, cognitive impairment, hot flushes, lymphedema, and psychological distress (41, 42). The influence of chemotherapy on sick leave duration is paramount (43).

#### Social Support at Work

Social support at work from colleagues and/or employer is likely to ease RTW and job retention (44–46). In Finland, women were shown to require more social support, mainly from occupational health services, than men after cancer (47). Lack of social support and discrimination in the workplace are barriers to RTW and job retention (34, 45, 48, 49). However, it is hard to disentangle women’s functional limitations after breast cancer from their feelings of discrimination at the workplace (50). Feelings of discrimination are mentioned in terms of undesired changes to the job, reallocation or reassignment, job stagnation, or deskilling (34, 50). A French study showed that women back at work after breast cancer perceived a lack of support from colleagues or employer (17 and 22%) and suffered from a feeling of being shunted aside (11%) (8). Discrimination at work increases job cessation by 10% in women after cancer, taking all forms together (50).

#### Work Adaptation

Beyond social support, work adaptation has a positive influence on RTW (51). However, a French survey showed that for 25% of cancer survivors their job was not being adapted (tasks and/or schedule) as desired (52). Despite the good RTW rate 3 years after breast cancer in France (82%), a study showed that 8% of women who returned to their previous job after breast cancer considered it inappropriate to their cancer-related symptoms, while 4% of those who had changed jobs within the same firm considered their new job inappropriate (8). A qualitative case study in France revealed great disparities in occupational adaptation after cancer, depending on the company’s or the patient’s strategies, with frequent underutilization of legal procedures intended to ease RTW and job adaptation for disabled persons (52). These results cast some doubt on stakeholders’ awareness or willingness regarding disability prevention and legislation. On the other hand, they underline the importance of exploring cancer survivors’ personal strategies for returning to work or not (34, 49, 53).

#### Information from Health-care Professionals

The paucity of appropriate information from health-care professionals about work resumption after breast cancer is mentioned in many qualitative studies as an obstacle to RTW (34, 45, 46, 48, 49). Health-care professionals act as if women should decide by themselves how and when to RTW, whereas women are in fact eager for counseling from them. Occupational health services could act as a resource on these issues (47), but occupational physicians (OPs) should provide better coordination and continuity of care in this respect (54).

#### Lack of Coordination

Lack of coordination between stakeholders is clearly mentioned in qualitative studies as an obstacle to RTW and job retention after disabling conditions (55–57) such as cancer (58). It was demonstrated, in Belgium, that different stakeholders followed different rules and objectives, with poor communication (58). In the UK, communication was identified as a key factor in easing RTW after cancer and even more in increasing job retention after return (59).

According to the synthesis of this evidence, RTW after breast cancer appears clearly as an intersectoral issue at the crossroads of the individual system of the patients, the health care, the workplace, and the insurance system. Furthermore, the RTW process and the work–life balance during and after the cancer journey can be thought of in a person–environment perspective, where different levels of the environment must be taken into account (interpersonal, organizational, and broader levels). Last but not least, social disparities in RTW and work retention after breast cancer were identified depending on the age, race, and education of the breast cancer patients. All these factors must carefully be taken into account when defining the logic model of the problem and the logic model of change of the intervention.

### Needs Assessment: Review of Indicators

As compared to the literature review, the indicators from data of the gray literature give a national and local estimation of the burden in the French context.

#### Magnitude of the Problem

The number of employed women who had a breast cancer in the Auvergne-Rhône-Alpes region amounted to 1,500 women (aged 25–54 years) and 2,000 (aged 25–59 years) in 2014 (60, 61). At the county scale of the Metropole de Lyon, the number of employed women possibly concerned by RTW issues after a diagnosis of breast cancer in 2014 was around 430 (women aged 25–54 years) and 565 (women aged 25–59 years).

#### Job Status

A proportion of 74.7% of breast cancer women who had a job at the time of their diagnosis remained employed 2 years later (62). The mean duration of sick leave for employed women after a breast cancer was 9 months. The mean duration from diagnosis to job loss after a breast cancer was 9 months (62).

#### Impact of Cancer in the Workplace (Cancer Survivors)

After cancer, women felt more penalized in their job than their male counterparts (13 vs 7.5%). Overall, 18% of cancer patients declared being stigmatized by their employer after their cancer, with a reduction of their responsibilities (43% of the persons feeling discriminated), of acquired advantages (32%), with career/salary stagnation (24%), downgrading (21%) or unsolicited hour accommodations (12%), or mutation (8%). At the time of returning to work after cancer, 47% of the workers declared difficulties associated with fatigue and side effects of treatments, 26% with cognitive limitations (attention and memory), and 6% due to their medical follow-up.

#### Impact of Cancer in the Workplace (Colleagues and Employers)

As regards the colleagues, 43% declared that the absence of a worker due to the cancer disturbed the workplace organization and 35% their own work organization. As regards the employers, 47% hired a temporary replacement worker until the return of their ill worker and 39% distributed the workload to the colleagues. Three employers out of four declared direct costs associated with the absence of a worker after cancer (63).

#### Job Retention Measures

All cancers considered, one worker out of three declared the absence of any support measure from the workplace during his/her cancer treatments, and one out of four declared the imposition of a less interesting job. No indicator could be identified about the proportion of patients benefiting from a pre-RTW visit with their OP. A great majority of employers (95%) agreed with partial sick leave to facilitate RTW after cancer, but only 49% were aware of the possibility for a worker to benefit from the status of handicapped worker after cancer.

#### Social Disparities in the French Context

Lower educated women had a higher risk of job loss 2 years after a diagnosis of cancer if their job was not accommodated (62). All cancers considered, the risk of job loss was increased by 21.6% for workers with a lower level of education and by 6.9% for workers in small businesses (62). The proportion of patients who did not disclose their cancer in the workplace was higher for lower educated workers (24%) and workers in small businesses (40%) as compared to all cancer patients (17%) (63).

### Needs Assessment: Qualitative Inquiry

The qualitative inquiry produced rich and varied results with a number of themes mentioned by the respondents, which will be published separately to allow a full description and discussion. The most salient results are summarized below.

#### Women Surviving Breast Cancer: Wanting a Better Work–Life Balance

Women with and after breast cancer had different motivations to RTW. These were related to financial issues, identity, social relationships, perceived utility in society, and the meaning of life. All the women had revised their personal priorities after cancer and wanted a better work–life balance. The possible timing of an intervention to help breast cancer patients resume work was carefully analyzed. The end of active treatments (chemotherapy and radiotherapy) was not mentioned by the participating women as an ideal timeframe. Rather, it was described as a golden period to take time for themselves before considering returning to work.

No, no, I want to be cool… and I don’t want to work full time any more. There is a time for everything. Enjoy life… When you could stay alive. (Breast cancer patient in a physical activity program)

The same period at the end of active treatments was paradoxically mentioned on several occasions as difficult for the women by health-care professionals.

As long as you are ill, there is a programme, your life doesn’t belong to you anymore. And one fine day, the doctor tells you: “It’s over, it’s all good. Thank you. Good bye (…)”. There is feeling of abandon that is extremely important. (Psychologist, in a rehabilitation center)

When they arrive here, they feel incapable of doing anything, they need help (…). They have been good little soldiers; they have done everything they were asked to do. And then they are told: “Here you are, it’s over, you need to go on.” And there they don’t have strength anymore. (Physiotherapist, in a rehabilitation center)

Early RTW was never mentioned as a desirable outcome. Conversely, some women reported they had resumed work too early and not satisfactorily due to persisting problems and the constraints of their medical follow-up. Other women mentioned that participating in a physical exercise program after their radiation therapy delayed their RTW, but increased the awareness of their limits, decreased their unrealistic expectations, and possibly led them to resume work in better conditions with greater chances of sustainability. Many women expressed feelings of anxiety provoked by the perspective of returning to work.

For me, it is not that cancer is marginal, but the big thing, that’s it, it’s work, the problem at work. (Breast cancer patient, in a rehabilitation center)

Important variations could be identified in the women’s intention to RTW, which were analyzed according to the stages of change model (64). Some women were not considering returning to work (pre-contemplative stage), while some others were weighting (contemplative) or preparing (action) this perspective. Some women had resumed work (maintenance) or had a new period of sick leave (relapse). To sum up, it was not possible to identify a consensus on the appropriate timing of an intervention after breast cancer, due to the variation between women and the fluctuation of their intention to resume work according to different stages of change.

#### Workplace Actors: a Three-Phase Process

Three important phases could be identified from the workplace perspective. During the sick leave, the absence of the worker had to be compensated, either by hiring another worker or by increasing the workload of the colleagues. The links between breast cancer women and their workplace during active treatments varied from a complete interruption to women keeping in touch regularly. Very few women continued working during their treatments. At the time of the first RTW, all the workplace respondents agreed on the lack of preparation between actors [breast cancer survivor (BCS), physicians, and employer], while coordination was deemed necessary. It was emphasized that both colleagues and management needed information and training to support a woman returning to work after cancer. Possibilities of the workplaces depended on their size and awareness. After the RTW, persisting problems were mentioned for the BCSs (fatigue, cognitive impairments), the colleagues (work overload, lack of support, and job termination of the replacement worker) and the management (dealing with confidentiality).

#### Health-care Professionals: Wondering How They Can Contribute to RTW

Oncologists had different levels of commitment toward the work issues of their patients. All declared having insufficient knowledge about work. They did not know how to help their patients when they expressed their concerns about resuming work. Oncologists mostly emphasized the importance of individual characteristics of the women (motivation and temper) in returning to work. They systematically underreported potential barriers such as depression, anxiety, cognitive limitations, and job requirements. They acknowledged the role of general practitioners (GPs) and OPs but could not precise in what respect. Oncologists from different medical specialties (medical oncologists, surgeons, and radiation therapists) reported collaborating rarely with each other and with other physicians (GPs and OPs). Female oncologists seemed more aware of RTW issues and barriers of their patients than their male counterparts.

When the chemotherapy is over, the radiation therapy is over, the surgery is over, and so, when the person is supposed to get back to a normal life, we consider that the job is done. (Medical oncologist)

The issue of return to work? Very frankly, we don’t discuss it, even with the social workers. (Medical oncologist)

For rehabilitation teams, physical activity programs after the radiation therapy were acknowledged as a period allowing the women to recover from a physical, psychological, and social point of view. RTW issues were rarely discussed during the programs. They were not considered as a priority and were left upon the women’s initiative. In agreement with patients’ views, rehabilitation professionals emphasized that delaying the RTW might lead to better results in terms of safety, quality of life, and sustainability.

Most of the women want to take time for themselves rather than returning to work. (Physiotherapist, rehabilitation center)

Still, they arrive at a time when everything is beginning, whereas for everybody, everything is over. Because it is then that something happens psychologically, when an emotional discharge may happen. (Psychologist, rehabilitation center)

General practitioners expressed that supporting their patients in returning to work was part of their role, but reported lacking of collaboration with social insurance physicians (SIPs) and OPs in this respect. Geographic disparities were mentioned, with patients having no access to occupational health services due to OPs shortage.

#### SIPs and Social Workers: Legal Constraints and Work Overload

Although all the SIPs felt concerned in helping women with breast cancer to RTW, they reported some limitations about their possibilities. Contrary to other medical conditions such as low back pain, they mentioned lacking guidelines to appreciate the risk of long-term work absence and disability after breast cancer. They had limited possibilities to exchange information with their social department due to legal constraints related to medical secrecy and privacy of personal data. However, some initiatives were taken at the regional and local levels of the social insurance agencies to better identify patients at risk of work disability, regardless of their specific medical condition.

The social workers of the social department of the national health insurance scheme (CNAMTS) had a clear mandate from their institution in work disability prevention. However, this mandate was only a little part of their other missions in the field of social work such as housing, education, autonomy of the elderly, etc. As a consequence, their possibilities in work disability prevention could be limited by work overload and lack of time. At hospital, social workers for the patients and their families shared their experience in different words. They reported difficulties to set standard criteria to identify socially deprived women. Rather, they insisted on the uniqueness of each situation and the need to consider the interactions of many factors such as financial resources, geographic isolation, social isolation, etc. Importantly, they expressed their reluctance to share information with OPs, by fear of negative consequences for the patients. As a consequence, they were also cautious about their advices to the patients regarding their OP.

### Data Integration: Preliminary Logic Model of the Problem

**Figure 1:**
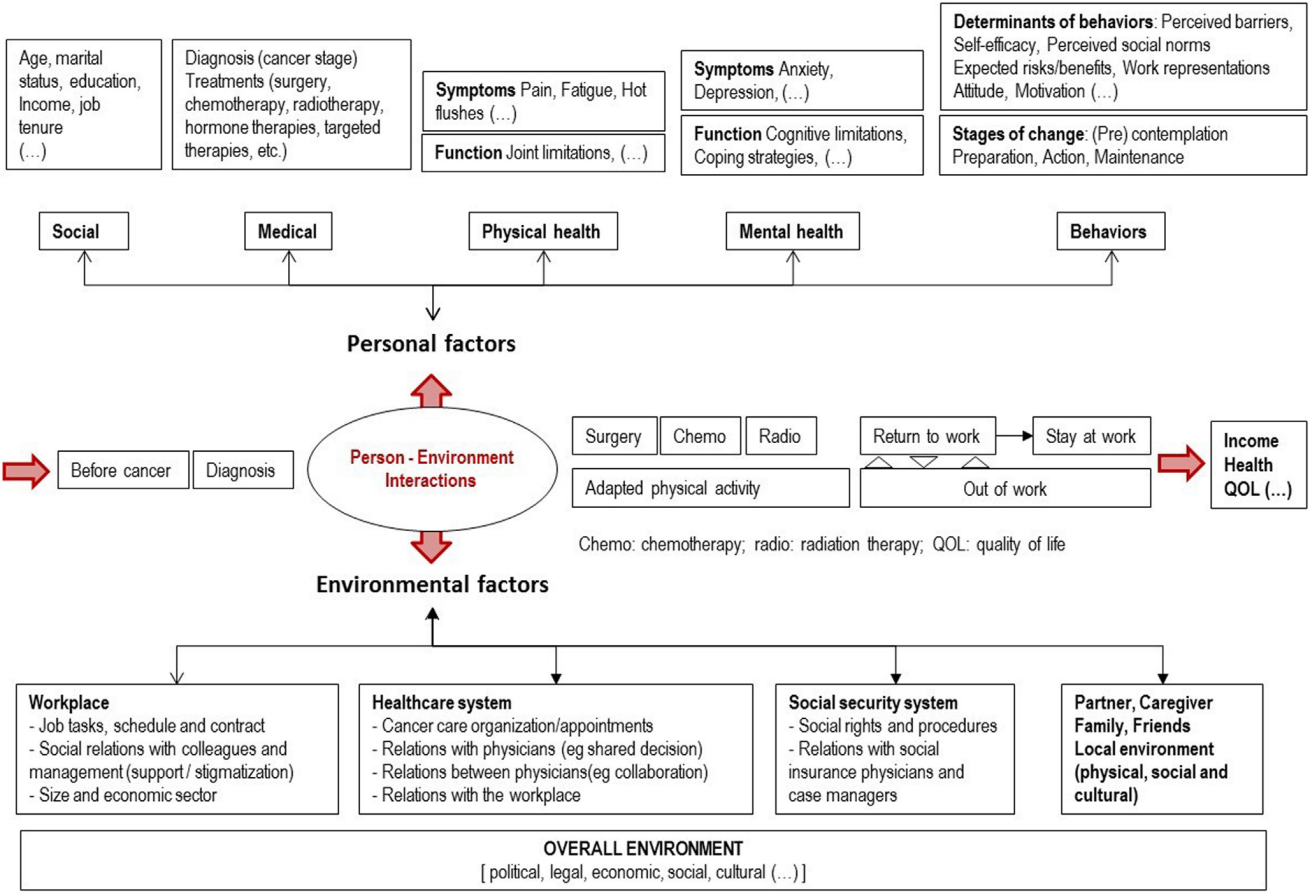
Logic model of the problem.

The results from the literature review, the search of indicators and the qualitative field inquiry led to the proposition of an overarching conceptual framework able to integrate various determinants associated with RTW after breast cancer (Figure 1). At the individual level of the breast cancer patients, the framework represents various dimensions (social and medical factors, physical and mental health, behaviors and their determinants, and stages of change) likely to influence the RTW process and outcomes. At the level of the environment, the factors likely to influence the process and outcomes are sorted into dimensions corresponding to the main categories of actors (workplace, health care, social security, and personal systems), within the overall environment at a more distal level (political, cultural, economic, etc.).

These factors at the personal and environmental levels are likely to interact at different time points in the cancer journey of breast cancer patients. The timeline is schematized by the main events along the cancer journey (before cancer, diagnosis, active treatments with surgery, chemotherapy and radiation therapy, physical activity, and after cancer with the RTW process and outcomes).

## Discussion

The interest of this needs assessment study is to describe the process and the results of the different steps (planning group, literature review, review of indicators, and qualitative inquiry) leading to a preliminary logic model of the problem of RTW after breast cancer.

### Summary of the Main Findings

#### Planning Group

The composition of the planning group represents faithfully the intersectoral nature of the problem. Despite the importance of the participative process in the IM protocol, only a few studies using this protocol in the field of cancer (29) and in the field of RTW (65) reported the association with a complete planning group from the needs assessment phase. This shortcoming has been discussed as a potential cause for the lack of relevance, acceptability, and/or efficiency of the studies developed (29). The elaboration of a charter to structure the partnership between the research team and the planning group is another original result, with no other example found in the literature on RTW.

#### Needs Assessment: Literature Reviews

The integration of results from cohort, qualitative, and intervention studies supported the formulation of an ecological view of the problem of RTW after breast cancer, which consequences are discussed below (preliminary logic model of the problem). Although this result was reported in previous studies (66, 67), it is worth mentioning since a majority of RTW interventions have focused on the individual level of BCSs rather than on their environment (15).

#### Needs Assessment: Review of Indicators

One main result of the review of indicators was to provide national and regional data to evaluate the magnitude of the problem. This is of particular importance in view to conduct the step 5 (implementation) and 6 (evaluation) of the IM protocol as regards the recruitment of the participants and the power calculation of the effectiveness study.

#### Needs Assessment: Qualitative Inquiry

The main finding of the qualitative inquiry is that “early RTW”, although largely advocated for in the field of musculoskeletal disorders (28), was not supported by the data. This is in line with previous studies outlining that women after breast cancer consider different matters before RTW, with mental preparation colored by uncertainty and vulnerability (68). Similarly, the variations of women’s point of view about RTW in this study are congruent with previous results showing that not all women change their view on life due to breast cancer (49). To our knowledge, it is the first time that the period following the active treatments is explicitly identified by BCS as a “golden hour” to take time for themselves, rather than to consider RTW. Paradoxically, the same period was described by several respondents as a “gap in the system” where women could be lost between the end of their active treatments and their RTW. An important implication in terms of RTW intervention is the necessity to adopt a tailored and responsive approach so as to adapt the propositions to each individual situation.

This finding supports the notion mentioned by many respondents that RTW should be viewed as a process, rather than a mere result. Once again, this echoes previous results that insist on the social and dynamic nature of RTW, with many interactions between the patient/worker and the actors of their environment at different phases of their cancer journey (58, 68). This result highlights the importance to adopt both a time-contingent basis and an ecological approach when developing an RTW intervention for women after breast cancer.

#### Preliminary Logic Model of the Problem

The conceptual framework elaborated from the data is not considered as the final version of the logic model of the problem for two reasons. In its current version, it is rather descriptive than explanatory, and hypothesis remains to be formulated about the direction, magnitude, and interactions of the many determinants on the process and outcomes of RTW after breast cancer. Second, this framework built by the researchers still needs to be discussed with the members of the planning group to make sure that it represents accurately their field experience.

This model presents the major interest to represent both an ecological view of the problem and to integrate the temporal dimension of the RTW process. As for the ecological view, BCSs are considered in their environment composed of various categories of stakeholders of the workplace, health care, insurance, and personal systems. This representation is in line with other “person-environment models” such as the arena model of Loisel et al. (28) and the Organizing model of practice for RTW in breast cancer developed by Desiron et al. after the International Classification of Functioning (67). Such models allow distinguishing more precisely different levels in the environment (micro, meso, and macro levels), corresponding to different determinants that may be targeted by an intervention (individuals, organizations, and wider structures of the political, legal, or economic context) (69).

As for the temporal dimension, the specificity of this model is to distinguish three main phases of the cancer journey: the phase of diagnosis, active treatments (surgery, chemotherapy, and radiotherapy), and after. RTW is considered both as a process and a possible outcome along this timeline, with the possibility of alternating periods at work and out of work, and eventually to stay at work or out of work in the long term. More distal outcomes are considered in line with the job status such as income (work-related), quality of life, health, or any other outcome of interest for the stakeholders. In this respect, the originality of this model is to avoid the risk of presenting RTW as the only desirable outcome. There is indeed the risk in the field of RTW research to reinforce a normative pressure toward RTW representing the global interests of the social protection system and the employers, whereas individuals may favor other choices than RTW and privilege a better work–life balance after breast cancer (35).

Overall, this model satisfies with the recommendations to (i) include psychosocial influences on individual’s cognitions and behavior (individual level) (ii) consider the relationship between the stakeholders (proximal environment); (iii) acknowledge the legal and political dimensions (wider environment); and (iv) consider the temporal dimension of the RTW process (70).

#### Social Disparities

Several dimensions of the model include factors associated with social disparities. The women’s position in the social hierarchy pertains to their social characteristics (e.g., education and income). Those factors are associated in the literature with other factors included in the logic model. As to the medical dimension, a lower SES is generally associated with a lower use of cancer screening services (71, 72) and a higher mortality (73). As to the determinants of behaviors, sociodemographic and economic factors influence the risk of weight gain after cancer, which may impact prognosis and risk of recurrence and of second cancer (74). As to the workplace environment, persons with a lower SES have physically heavier jobs with lower levels of autonomy and more temporary contracts (13, 75). As to the health-care system, they have a poorer access to specialized and standardized care (76). Social support is a key point to explain inequalities in waiting times between the first imaging procedure and the first treatment (77). There is qualitative evidence that socially deprived young women face many barriers and have unequal access to supportive care services after breast cancer (78). As to the social protection system, persons with a lower SES are more frequently concerned by non-take-up of their social rights, either by lack of information, demand, or attribution (52). In their personal environment, they usually have a lower social capital and are more frequently isolated socially and/or geographically. Finally, women with a lower SES are more likely to be on long-term sick leave and to lose their job after a breast cancer (9). The integration of these factors in the logic model of the problem allows formulating causal hypothesis about the possible mechanisms by which social disparities in employment after breast cancer take place. In the following steps of the IM protocol, this should allow developing an intervention with a greater cultural relevance, targeted to specific factors that contribute to social disparities. As an example, the intervention might propose social counseling to limit non-take-up of social rights, associated with a psychological approach to increase self-efficacy and/or a workplace component to ease job accommodation.

### Strengths and Limitations

The first strength of this study pertains to the criteria adopted to ensure a genuine participative process between the research team and the planning group. This is a key feature of the IM protocol which importance is paramount to benefit from the field experience of the stakeholders. The composition of the planning group reflects the intersectoral nature of work disability prevention theorized in the arena model (28). Another strong feature of this study is the detailed description of the needs assessment with different data sources. The integration of the results allowed building an innovative logic model of the problem. Although preliminary, this model brings together an ecological view and a time-contingent specific perspective on the RTW process after breast cancer. It is expected to lead to a better understanding of the causal mechanisms of RTW outcomes along the timeline and to help develop an intervention that is culturally relevant to the needs of the different stakeholders.

Some limitations of the study pertain to the sampling of the respondents who participated in the qualitative inquiry. We cannot exclude a selective participation since most participants were found *via* personal connections of the researchers and snowball sampling. A possible consequence might be the underrepresentation of divergent point of views and the underestimation of barriers. However, the triangulation with other data sources (literature review and indicators) is likely to minimize this consequence in the case of a participation bias. The inquiry among institutions could not be done as expected due to bureaucratic complexity and some refusals to conduct individual interviews and FGs. This barrier could not be removed and the needs of the institutional actors could not be explored in their own view as it was the case for the other categories of actors. In this respect, it was not possible to triangulate the patients’ point of views as regards the complexity of the social protection system and their difficulties to navigate the system (79). Also, despite important efforts, we included only two socially deprived women with breast cancer. This difficulty was considered as an important barrier to anticipate regarding the development of the future intervention and the way to reach lower educated persons. A possible strategy to remedy this shortcoming could be to spend more time in the field with associations and/or social workers to gain their trust and reach socially deprived persons.

### Recommendations for Future Research

It is recommended from this study to investigate possible strategies to better involve socially deprived persons, and stakeholders from institutions. It is recommended to develop a transdisciplinary theoretical perspective to expand the potential of the preliminary logic model of the problem. It is recommended to investigate the interest and limitations of a charter of partnership to structure the collaboration between researchers and stakeholders in work disability prevention. Finally, it is recommended to investigate the relative advantage of rapid methods of needs assessments to render this step less time-consuming.

### Next Steps of the FASTRACS Project

Several steps need to be carried out to complete the tasks of the needs assessment phase of the FASTRACS project. It is planned to define and adopt formally a charter of partnership by means of a Delphi consensus process associating the members of the planning group and the research team. The first version of the logic model of the problem proposed by the researchers (Figure 1) needs to be discussed with the members of the planning group to make sure it represents faithfully their experience of the problem. Last but not least, the objective of the intervention needs to be defined before moving to the next step of the IM protocol, that is, defining the logic model of change. It is expected to complete the step 2 (logic model of change), step 3 (theoretical change methods and their applications), step 4 (program plan), and step 5 (implementation) by August 2018. The evaluation step of the intervention is planned by means of a randomized controlled trial.

## Conclusion

The IM protocol was used for the first time in France to develop an RTW intervention after breast cancer. The needs assessment tasks were carried out carefully and took longer than expected. Although the process was time-consuming, its results form a crucial basis for the intervention that will be developed. The logic model of the problem integrates actors of the workplace and health-care environment, contrary to previous interventions which were ineffective. A special emphasis was put to structure the participative planning group and its input to the FASTRACS project in the long run. The next steps of the IM protocol will be followed to develop, implement, and evaluate the FASTRACS intervention.

## Ethics Statement

This study was carried out in accordance with the recommendations of the Comité de Protection des Personnes Sud-Est II with written informed consent from all subjects. All subjects gave written informed consent in accordance with the Declaration of Helsinki. The protocol was approved by the Comité de Protection des Personnes Sud-Est II (IRB no 00009118).

## Author Contributions

All the authors brought substantial contributions to the conception or design of the work or the acquisition, analysis, or interpretation of its data. All the authors revised the work critically and approved its final version to be published. They all agree to be accountable for all aspects of the work.

## Conflict of Interest Statement

The authors declare that the research was conducted in the absence of any commercial or financial relationships that could be construed as a potential conflict of interest. The reviewer FS declared a past co-authorship with one of the authors J-BF to the handling editor. The reviewer AR and the handling editor declared their shared affiliation.
