# Supplementary material for: Developing a Return to Work Intervention for Breast Cancer Survivors with the Intervention Mapping Protocol: Challenges and Opportunities of the Needs Assessment
Source: Front Public Health. 2018 Feb 23;6:35. doi: 10.3389/fpubh.2018.00035 (PMC5829033; doi:10.3389/fpubh.2018.00035)
Supplement: Supplementary file 1 [file Table_1.DOCX]

**Supplementary material:** Themes and location of the interviews

| **Stakeholders** | **Patients** | **Healthcare professionals** | | | **Institutions** | **Workplaces** |
| --- | --- | --- | --- | --- | --- | --- |
|  |  | Rehabilitation teams | Oncologists | General practitioners |  |  |
| **Data collection** | 3 FG + 10 INT | 3 FG | 20 INT | 3 FG | 3 FG | 18 INT |
| **Location** | Rehabilitation centre (FG and 1^st^ INT) ; at home (2^nd^ INT) | Rehabilitation centre | Cancer care centre | General medicine department, University | Institution (hospital, social work department) | Workplace |
| **Themes** | Current physical and mental health; family situation | Physical and mental health evolution of BCS during rehabilitation | Physical and mental health of BC patients (based on one or two illustrative clinical cases) | Physical and mental health of BC patients |  | Representations of breast cancer |
|  | Physical activity program: perceived benefits and utility; unmet needs to RTW | Physical activity program: perceived benefits and utility; | Side effects of cancer and treatments |  | Organizational (institutional) commitment, procedures and support, during sickness absence, work re-integration and after RTW | Organizational (workplace) commitment, procedures and support, during sickness absence, work re-integration and after RTW |
|  | Cancer care pathway: relations with and between healthcare professionals | Relations with BCS; personal commitment | Relations with BCS; personal commitment | Relations with BCS; personal commitment | Personal experience with RTW of BCS | Personal experience with RTW of BCS |
|  | Relations with different healthcare professionals (oncologist, general physician, occupational physician, social insurance physician) | Relations with other healthcare professionals | Relations with other healthcare professionals | Relations with other healthcare professionals | Relations with healthcare professionals | Relations with healthcare professionals |
|  | Job tasks and job satisfaction prior to the sick leave | Return to work: perceived personal role | Return to work: perceived personal role | Return to work: perceived personal role | Return to work: perceived personal role | Return to work: perceived personal role |
|  | Return to work: perceived needs to RTW, perception of “good RTW”, personal agenda, perceived support from workplace actors during treatments, disclosure of the diagnosis | Return to work: perceived needs of BCS to RTW ; specific needs of women with low SES | Return to work: perceived needs of BCS to RTW ; specific needs of women with low SES | Return to work: perceived needs of BCS to RTW ; specific needs of women with low SES | Return to work: perceived needs of BCS to RTW ; specific needs of women with low SES | Return to work: perceived needs of BCS to RTW ; specific needs of women with low SES |

INT= interview; FG= focus group; BCS=breast cancer survivor; RTW=return to work; SES=socioeconomic status
