# Supplementary material for: Developing a Return to Work Intervention for Breast Cancer Survivors with the Intervention Mapping Protocol: Challenges and Opportunities of the Needs Assessment
Source: Front Public Health. 2018 Feb 23;6:35. doi: 10.3389/fpubh.2018.00035 (PMC5829033; doi:10.3389/fpubh.2018.00035)
Supplement: Supplementary file 2 [file Table_2.DOCX]

**Supplement 2:** Comparative table of the values, needs and commitments of the different stakeholders

These data were collected during the first meeting of the planning group with the research team, by means of written answers that were typed and analyzed qualitatively. The next step of the process will be to formulate propositions based on these values, needs and commitments, to elaborate the charter of partnership,. The propositions elaborated by the research team will be validated by the planning group by means of consensus (Dephi method). The process and the result of the charter of partnership will be published.

| **STAKEHOLDERS** | **Patients & associations** | **Employers** | **Healthcare professionals** | **Institutions** | **Researchers** |
| --- | --- | --- | --- | --- | --- |
| **VALUES** |  |  |  |  |  |
| **Solidarity** | Solidarity | Solidarity | Needs | Solidarity | Mutual help |
|  | Relief the burden of BCS |  | Frailty |  |  |
|  |  |  | Isolation |  |  |
|  |  |  | Support |  |  |
| **Support** | Support |  | Support the worker in social and occupational issues | Global support | Support |
|  |  |  | Support BCS to resume a normal life with goof quality of life |  |  |
| **Trust** | Trust |  | Trust | Mutual trust | Mutual trust |
| **Respect** | Respect | -Diversity (accept differences, accept frailty) |  |  | Respect |
|  | Confidentiality |  |  |  | Confidentiality |
|  | Confidence |  |  |  |  |
|  | Being non jugemental |  |  |  |  |
| **Collaboration** | Make links | Shared project | Links | Multidisciplinarity | Collaborations, links, partnership |
| **Exchanges** | Listening |  | Exchange | Share | Dialogue |
|  |  |  |  | Share with all the persons around the BCS |  |
| **Excellence** |  | Requirement | Scientific outreach |  | Scientific excellence |
| **Accountability** | Fair and real evaluation |  |  | Transparency | Sincerity |
| **Health** | Make BCS responsible for their health |  |  | Work as a determinant of health | Work good for health |
| **Work** |  | Work | Job retainment |  |  |
| **Person-centered approach** | Promote self-care |  | Patient-centered (global) approach | Develop a global approach of health | The individual |
|  |  |  |  | Consider all the dimensions of the person |  |
| **Social utility** | Real social utility |  | Social benefits | Social utility; greater good | Social utility; |
| **(…)** |  |  |  |  |  |
| **NEEDS** |  |  |  |  |  |
| **Collaboration** |  |  | Collaboration | Links between partners | Collaboration |
|  |  |  | Create a network | Importance of pluridisciplinarity | Partnership, shared decisions |
|  |  |  | Work together with institutionals | Reinforced partnership to face complexity |  |
|  |  |  | Improve multidisciplinary care (social workers, psychologists, occupational therapists, etc.) | Teamwork; collective thinking |  |
| **Communication** | Exchange | Communicate on RTW | Better anticipation and communication between healthcare and work | Fluidity | Constructive exchanges |
|  |  |  |  |  | Share |
|  |  |  |  |  | Understanding, information, communication |
| **(…)** |  |  |  |  |  |
| **COMMITMENTS** |  |  |  |  |  |
| **Collaborate** | Collaboration |  | Use networks | Make partnerships and work together differently | Coordinate and bring the project to its end |
|  | Bring partners together |  | Participate to the exchanges |  |  |
| **Communicate** | Relay information |  | Relay information | Communication support |  |
| **Personal implicattion** | Personal implicattion |  | Personal implicattion | Personal availability | Assiduity |
|  |  | Share testimonies (case studies) | Testimonies | Logistic support | Maintain the framework |
|  |  |  | Involve new participants (patients, workplaces) | Change support | Maintain the scientific rigor |
| **(…)** |  |  |  |  |  |
